# Organizational Emotional Capability Perspective: Research on the Impact of Psychological Capital on Enterprise Safety Performance

**DOI:** 10.3389/fpsyg.2022.854620

**Published:** 2022-04-20

**Authors:** Cheng Peng, Ke Xue, Yue Tian, Xuezhou Zhang, Xi Jing, Haolun Luo

**Affiliations:** ^1^International Business School, Yunnan University of Finance and Economics, Kunming, China; ^2^OCT Yunnan Cultural Investment Group Co., Ltd., Kunming, China

**Keywords:** managers’ psychological capital, organizational emotional capability, positive psychology, safety management, safety performance

## Abstract

Theoretical researchers of manager psychology have excellent potential to extend its research framework to more enterprise application areas, such as innovation, performance, and safety in production. Research in these areas has also been increasing in the past 10 years. Psychological capital is composed of four aspects: self-efficacy, hope, optimism, and tenacity. It plays an essential role in stimulating organizational growth and improving organizational performance. In safety management work, managers, as the core members of the organization, have a relationship between their psychological capital and employees’ safety performance. Nevertheless, the closeness of the relationship between psychological capital and employee safety performance has not been fully demonstrated by academic circles. Based on positive psychology theory, this paper conducts a questionnaire survey of 157 managers and 314 employees related to safety work in manufacturing enterprises. From the new perspective of organizational emotional capability, this paper investigates the complex and extensive social-psychological role in organizations and combs, analyzes, and integrates relevant psychological research to construct the influence mechanism of managers’ psychological capital and employee safety performance. Finally, the three important issues found based on data analysis were: (1) Managers’ psychological capital has a significant positive impact both on employee safety performance and organizational emotional capability; (2) Organizational emotional capability has a significant positive impact on employee safety performance; (3) organizational emotional capability plays a partial mediating role in the relationship between managers’ psychological capital and employee safety performance.

## Introduction

In the production process of enterprises, safety is a significant issue. While manufacturing enterprises such as the construction industry and the tobacco industry have promoted economic development, they have also been added to the list of the most dangerous industries due to their complex processes, high personnel mobility, and frequent emergencies (Fang et al., 2015). Long-term uncertainty in the workplace is likely to cause serious crises and conflicts in the future (Chin et al., 2021). In the construction process, there are often many risk factors that affect production. These factors include the following: (1) the relevant staff lacking the professional quality and safety awareness required by the construction industry and (2) the management not carrying out effective and comprehensive safety management of workers. These situations lead to frequent safety problems. In the production process of tobacco companies and other industrial companies, many employees have a one-sided understanding of safety management. Managers lack adequate supervision and management mechanisms for employees, which leads to habitual violations of regulations by employees of the company. The production and operation of enterprises have brought severe safety hazards. There is a significant negative correlation between employee safety performance and safety accidents, and good safety performance has a significant impact on reducing safety accidents in production companies.

It is critical to encourage manufacturers to detect risks in time, and the existing early warning model is not reliable enough to warn companies of potential risks (Duan et al., 2021). In the early stage, injuries and accidents mainly measured enterprise safety performance. Guo and Yiu (2016) due to a specific lag effect, this measurement scheme cannot provide early warning information for safety accidents. Because of this, Neal et al. (2000) introduced the concepts of task and relationship performance to safety management based on the research theory of scholars such as Borman and Motowidlo (1993). They propose dividing safety performance into safety compliance and participation behavior. Research has shown that employees’ safety compliance and safety participation behaviors have a significant impact on the occurrence of safety accidents (Gao et al., 2015). Traditional employee safety performance management focuses on accidents, injuries, and deaths caused by certain unsafe behaviors of employees during the work process and then adopts aggressive reinforcement measures to solve these problems, such as loud warnings, scolding, and deductions of wages or bonuses. It warns employees of unsafe behaviors, but these methods can only serve as warnings and cannot allow employees to establish safety concepts and consciously abide by safety regulations and may even lead to employees’ rebellious psychology. For the above reasons, safety accidents in industrial and mining enterprises frequently occur worldwide. On December 21, 2020, the Chinese emergency management department published an investigation report on security incident cases. One of the most influential accidents was the water inrush and mud inrush accident in Anshi Tunnel of Yunfeng Expressway in Fengqing County, Lincang City, Yunnan Province on November 26, 2019. The construction project caused 12 deaths and ten injuries due to improper construction. The direct economic loss reached 25.2501 million yuan; according to International Labor Organization statistics, 2.78 million people die from occupational accidents every year, and approximately one-sixth of fatal accidents occur in the construction industry (Zhang et al., 2019). Therefore, looking for a new safety management model has become one of the significant problems that production enterprises must solve.

At present, with the vigorous development of positive organizational behavior management, safety performance research ushered in a new opportunity. Scholars focus on how to take positive leadership measures to give play to the advantages and potential of employees. For example, from employee evaluation, the influence of ethical leadership on employee safety performance is studied. Subsequently, the influence of managers’ psychological capital on employee behavior is gradually carried out. Based on positive psychology theory, Luthans and Youssef (2004) and Luthans et al. (2006) believe that psychological capital includes four aspects: self-efficacy, optimism, hope, and resilience. Psychological capital is an essential means for companies to gain a competitive advantage. Psychological capital focuses on developing and maintaining the individual’s positive psychological state, positively impacting individual behavior and organizational performance (Ke et al., 2009a). Because of the difference in status and power, managers are more likely to imitate employees. Managers with good psychological capital will set a positive example for employees. Employees will not only imitate the behavior of managers but also follow the positive attitude of managers, such as the self-confidence, optimism, and tenacity of model managers. These attitudes increase employees’ positive psychological states, help them maintain a positive attitude toward the measures taken by the management, and allow them to be more receptive to the safety measures of the enterprise, thus affecting the safety behavior and safety performance of employees.

Studies of psychological capital, organizational emotional capability, and safety performance have started worldwide. Nevertheless, most research focuses on employees’ psychological capital influencing safety performance, emotional capacity influencing innovation and organizational performance. There is no link between the practical study, nor does it focus on the psychological connection between managers and employees. Therefore, based on construction, tobacco, and other industrial enterprises in China, from the perspective of organizational emotional capability, this paper takes managers and employees in construction, tobacco, and other industries as the research object and studies the influence mechanism of managers’ psychological capital and employees’ safety performance. In addition, a mediating variable, organizational emotional capability, is introduced to explore and analyze how management psychological capital influences employee safety performance through organizational emotional capability to clarify the influence path of management psychological capital on employee safety performance.

## Literature Review and Hypothesis

### Definition of Concepts

The concept of psychological capital originates from positive organizational behavior and positive psychology. It is a positive psychological state that individuals show in the process of growth (Luthans, 2002; Luthans and Youssef, 2004; Luthans et al., 2006). Luthans F. L. et al. (2007) developed a psychological capital scale based on four dimensions of psychological capital and a total of 24 measurement items. This research also found that psychological capital may be the key to better understanding employee occupational stress (Avey et al., 2009). In the same period, Ke et al. (2009b) developed a psychological capital scale with Chinese characteristics and believed that the concept of psychological capital has a second-order two-factor structure: (1) transactional psychological capital and (2) interpersonal psychological capital. Transactional psychological capital is similar to the psychological capital measured by the scale of Luthans scholars. In recent studies, Luthans F. et al. (2007) reviewed previous theoretical mechanisms, antecedents, results, and applications of psychological capital and proposed new psychological capital research directions, such as video games and gamification technology.

Safety performance refers to the number of accidents and injured people (Zohar, 2000). Safety performance can measure the results of employee behaviors under the control of organizational risks, and reflects the effect of the implementation of organizational safety objectives and policies (Gao et al., 2017). Research on safety performance, such as Neal et al. (2000), according to Borman and Motowidlo’s (1993) task performance and relationship performance theory, introduces the concept to the field of security and proposes that behavior can be divided into safety compliance and safety performance. In earlier studies, Neal et al. (2000) also divided safety performance into three dimensions based on the criterion of behavior perspective, adding the behavioral dimension of safety awareness. With the gradual deepening of research on safety performance, scholars continue to expand the dimensions of safety performance classification according to specific situations. Vinodkumar and Bhasi (2010) studied the impact of safety management on safety behavior through empirical analysis of 11 measurement items and concluded that the improvement in safety performance is of great significance to the development of enterprises. Safety performance is measured in different ways, including leading and lagging. Traditionally, the construction industry generally uses lagging indicator such as accident rate and casualty rate to measure safety performance (Hinze et al., 2013). These methods are applicable mainly because they are easy to collect and can be used for trend identification (Lingard et al., 2013). The leading indicator can give appropriate feedback to safety management before accidents occur for improvement (Hinze et al., 2013). For example, operational error may be a leading metric for which the researchers established a metric (Mohaghegh and Mosleh, 2009).

Organizational emotional capability is an organization’s ability to perceive, understand, monitor, adjust, and use organizational emotional resources to guide employees to express their emotions in its workflow (Akgün et al., 2007). As shown in the literature, most of the current research on organizational emotional capability involves organizational innovation performance and organizational security. Huy (1999) proposed a six-dimensional structural framework of organizational emotional capability based on individual emotional states and explained the connotation of emotional dynamics at each level in the framework. These connotations are harmony dynamics based on sympathy expression, experience dynamics based on empathy expression, identity dynamics based on love expression, game dynamics based on interest stimulation, free expression dynamics based on actual emotion stimulation, and inspiration dynamics based on hope stimulation. Akgün et al. (2009) used 18 measurement items from the above six dimensions to investigate industrial enterprises in Turkey and found that enterprises’ emotional capacity affects their financial and market performance through their innovation capacity.

### The Impact of Managers’ Psychological Capital on Employees’ Safety Performance

Employees’ human capital, social capital, and psychological capital have gradually become a positive force to improve the competitiveness of employees and enterprises in enterprise development processes. Studies show that collective psychological capital also has a particular impact on enterprise performance (Chen et al., 2021). Therefore, psychological capital is essential for enterprises to obtain competitive advantages. At present, most scholars’ research on psychological capital mainly focuses on two themes: (1) The first topic is the influence of psychological capital on “antecedent variables,” such as individual factors (Jung and Yoon, 2015), leadership and intervention factors. Researchers believe that transformational leadership (Lei et al., 2020), ethical leadership (Bouckenooghe et al., 2015), authentic leadership (Malik and Dhar, 2017), empowerment leadership (Park et al., 2017), and other variables have a particular impact on employees’ psychological capital. (2) The second theme is the impact of psychological capital on “outcome variables.” Most of the current research on the “outcome variables” of psychological capital focuses on the influence of employees’ psychological capital on personal attitude, behavior, and performance. Psychological capital is positively correlated with ideal employee attitudes and negatively correlated with bad employee attitudes. Employees with high psychological capital report an optimistic attitude at work, believe they can achieve success, are willing to accept challenges in work and are not afraid of difficulties in work. In the face of stress at work, employees with a higher level of psychological capital also have a higher level of safety participation (He et al., 2019).

Current research on employee safety performance mainly focuses on two dimensions: enterprise and individual. From the perspective of enterprises, it is found that the adequacy of safety equipment and facilities provided by enterprises is crucial to the safety performance of employees (Ragona, 2019). Unsafe behavior in the construction industry is the most critical factor leading to workplace accidents. Research of the construction industry found that first aid training positively impacts employees’ safety practices (Ali et al., 2021). Therefore, it is necessary to provide relevant safety training for employees before they start work, which can make them realize the importance of mastering safety knowledge and complying with safety behaviors to reduce safety risks and improve the safety performance of employees (He et al., 2019). Especially for manufacturing enterprises, safety prevention behavior is more important for improving safety performance (Hong et al., 2018). From the perspective of individuals, the research mainly focuses on the impact of safety attitudes on safety performance. The safety level of an enterprise in a certain period is determined by the safety behaviors of its internal employees, and their safety attitude largely depends on how individuals perceive the risk of accidents. Therefore, when communicating information, managers should not only urge employees to complete their work tasks but also educate employees to be responsible for their own and colleagues’ safety (Su, 2021). If employees suffer from job burnout and negative emotions, it will reduce their safety performance; in contrast, employees’ favorable safety attitudes and behaviors positively impact safety performance in safety practice (Kundu et al., 2016).

According to social learning theory, managers’ positive psychological qualities are often learned and imitated by their subordinates through examples and demonstrations to form positive psychological qualities and stimulate their positive behaviors (Liu et al., 2015). Managers’ psychological capital can be transmitted to subordinates through the infection effect (Walumbwa et al., 2010). Studies show that employees with a high sense of innovation self-efficacy engage in positive, creative behaviors when provided with a supportive innovation environment (Jaiswal and Dhar, 2015). There is a significant positive correlation between employees’ psychological capital and job performance (Rabenu et al., 2017). Managers’ positive reactions and supportive actions directly and immediately impact employees’ safety behaviors (Fang et al., 2015), and their psychological capital has a positive impact on innovation behavior (Qiu et al., 2015). Under production pressure, managers may not prioritize safety factors, and even encourage employees to take shortcuts to meet the production plan, thus reducing the safety performance of employees (Guo and Yiu, 2016).

Moreover, people with higher levels of psychological capital also have higher levels of resilience, enabling them to cope better with stressors and prevent distress and depression. Highly optimistic people have positive expectations for the future (Min et al., 2015), and the positive role of managers strongly affects the safety compliance behaviors of their subordinates, thus affecting the safety performance of employees. Nykänen et al. (2019) found through a questionnaire survey of vocational school students that self-efficacy influences safety performance. Based on the above theoretical basis, this paper proposes the first hypothesis:

**H1:** Managers’ psychological capital positively affects employees’ safety performance.

### The Impact of Organizational Emotional Capability on Employee Safety Performance

Studies on organizational emotion show that although emotion is an internal psychological experience, it has the dual characteristics of psychology and sociology. It can act on organizations just as it does on employees, thus affecting employees’ organizational assumptions or basic judgments about the organization (Hareli and Rafaeli, 2008). At present, the research on organizational emotion is still in the preliminary stage. Through literature review, it is found that scholars mainly study the concept of organizational emotional capability from three perspectives: resource perspective, emotion perspective, and stimulus-response perspective. The research on organizational emotional capability focuses on resources, which can be reflected in two theories, namely resource-based theory (RBT) and Conservation of Resource Theory (COR). Emotion is an essential organizational resource. It has the characteristics of value, scarcity, irreplaceability, and hard to imitate, and can be transformed into other resources or play a role in the organization’s development. Weiss and Cropanzano (1996) put forward the theory of emotional events, arguing that there are two ways in which emotional responses to behaviors. One is called emotion-driven behaviors directly. The other is to influence behavior through attitude, called judgment-driven behavior. From the theoretical perspective of stimulation-response, the cognitive process of an organism is a mental activity in which the organism actively obtains and processes stimuli. For the first time, Li et al. (2017) regarded organizational emotional capability as resource stimulation in organizational resource transformation. They realized corporate innovation through cognitive identification activities such as organizational commitment.

Since there are few studies on the “antecedent variables” of organizational emotional capability, this paper will not review them. The research on the “outcome variables” of organizational emotional capability mainly focuses on innovation ability, innovation performance, and organizational performance. Arias-Pérez et al. (2019) found that organizational emotional capability has a particular impact on enterprise innovation performance through their research on middle-low technology SMEs in Colombia. Akgün et al. (2007) surveyed local enterprises and found that organizational emotional capability, which takes free expression, experience, harmony, and identity dynamics as internal measurement dimensions, significantly impacts product innovation performance. Shanker et al. (2017) studied Malaysian enterprise managers and found that managers’ innovative work behavior plays an intermediary role in the influence mechanism between organizational innovation climate and organizational performance. Nugroho et al. (2020) found that the organization’s incentive dynamics can stimulate employees to find reasons for difficulties and find solutions, which can further improve organizational performance. Fang et al. (2015) found that organizational identity dynamics can provide employees with a sense of belonging, promote the formation of emotional bonds among employees to enhance their sense of identity, and enhance the willingness to cooperate and enthusiasm for safety participation among employees, thus improving their safety performance. In a hostile organizational environment, employees cannot gain a sense of identity and belonging, which reduces employee collaboration behavior in the organization (Duan et al., 2022). Sadia et al. (2016) found that an organization’s harmony dynamics and experience dynamics represent harmonious working relationships, good communication, and mutual trust between employees, and this environment can improve workers’ safety performance. In addition, good communication between on-site managers and employees can increase the sense of trust of employees, can effectively convey safety awareness to employees, and help employees understand safety rules to improve the safety performance of employees. The organization’s game dynamics encourage members to dare to try and take risks. In this environment, employees do not cover up decisions that fail due to a lack of intention (Nutt, 2004). Employees can also point out safety management issues found in work management at any time to improve employee safety performance. The “dynamic nature of organizational freedom of expression” means that companies encourage employees to express various genuine emotions legitimately without fear of punishment or criticism (Akgün et al., 2009). Rational expression of emotions meets the emotional needs of employees and promotes their work enthusiasm, thus improving organizational performance. Based on the above theoretical basis, this paper proposes a second hypothesis:

**H2:** Organizational emotional capability has a positive impact on employee safety performance.

### The Influence of Managers’ Psychological Capital on Organizational Emotional Capability

Due to the high competitive pressure and the accelerated market change in modern enterprises, an increasing number of enterprise managers realize that employees’ work pressure and ideological burden are increasing. They are more concerned about the mental health of their employees while focusing on compensation, benefits, and growth. Employees can accept the reality of heavy work and pay cuts but desperately need psychological comfort. With the development of science and technology, the complexity of organizational work has increased, and the study of emotional work has gradually expanded from personal psychological experience to social situations. Enterprise employees are in a collective working state, and the organization is composed of people with various emotions. As a result, personal emotions permeate every aspect of an organizational workplace and become an essential part of organizational life. RBT regards organizational emotional capability as a scarce resource and holds that organizational emotional capability is a unique resource that is difficult to imitate and replace (Akgün et al., 2009). He et al. (2017) conducted an empirical analysis from the perspective of leader-member exchange theory based on resource-based theory and found that perspective-taking between leaders and members has a significant positive impact on organizational emotional capability. Organizational emotional capability comprises six levels of emotional dynamics, and the levels of emotional dynamics shown vary with emotional resources. Psychological capital is composed of four aspects, among which managers with optimistic personalities can convey hope, joy, and positive emotions to the organization. Managers with a high sense of self-efficacy are calm in work and good at challenging and infusing the spirit of trial and error into the organization. Transformational leaders have a strong sense of self-efficacy, an optimistic work attitude, and the ability to face a complex work environment. Studies have found that transformational leaders significantly positively impact organizational emotional capability (Zineldin, 2017). Based on the above theoretical basis, the third and fourth hypotheses are proposed in this paper:

**H3:** The impact of managers’ psychological capital on organizational emotional capability.

**H4:** organizational emotional capability plays a mediating role in the relationship between managers’ psychological capital and employees’ safety performance.

## Research Design

### Data Collection and Sample Survey

In this paper, the data collection form is the questionnaire survey. The questionnaire distributed object for some of China’s industrial enterprises contains construction, tobacco and mining, and other enterprises, mainly construction enterprises and tobacco. The subjects of the questionnaire survey were grassroots managers and front-line employees closely related to safety work in their enterprises. Due to the particularity of the construction industry, the working cycle of front-line employees is usually from project initiation to completion of the whole construction cycle, so the hard-working environment and long-term absence from the home affect the mentality of employees. Therefore, as the main body of wealth creation in construction enterprises, the safety performance of front-line employees is significant. Basic-level managers are subservient to high-level and middle-level managers. Their primary responsibility is to assign specific work tasks to front-line staff, command and supervise on-site operations, and ensure the practical completion of various work tasks. Grassroots managers work with employees most of the time, and their personalities, behaviors, and work situations play an imperceptible role in the psychological perception of employees. Effective communication between grass-roots managers and employees and a good organizational atmosphere between them can play an important role in improving employees’ safety awareness and strengthening enterprise safety management. Therefore, grassroots managers and their direct employees are selected as the subjects of this questionnaire survey, which fits the theme of this study. In this study, the number of managers and employees was 1:2 to distribute questionnaires in pairs (before issuing questionnaires, it was assumed that one manager supervised two employees). To reduce common variance, the paired samples of managers and subordinates are used for data sampling analysis. To ensure the quality of the questionnaire survey results and avoid the influence of certain contingency in the sampling process on the research conclusions, at the same time, due to the limitation of time, human resources and other resources required by the questionnaire survey, the ratio of managers and subordinates in the questionnaire survey is 1:2. Before issuing questionnaires, the research team asked for the consent of enterprises by elaborating the purpose of this questionnaire survey and committed to the anonymity and confidentiality of the questionnaire. Before issuing the questionnaires, the research team numbered the questionnaires from different enterprises to ensure the integrity of the information collected. To ensure the rationality and effectiveness of the formal questionnaire, 50 questionnaires were issued for a small sample test before the formal survey, and the questions reflected in the test results were adjusted before the formal questionnaire survey. Questionnaires in the formal stage were collected in the form of a questionnaire star and via on-site distribution. The research team distributed 480 questionnaires to employees and 200 to direct managers. A total of 168 questionnaires for managers and 365 questionnaires were collected. After removing invalid data demonstrating excessive answer consistency or with more than three missing values, 157 valid questionnaires for managers and 314 valid questionnaires for employees were obtained.

The effective recovery rate of the managers’ questionnaires was 78.5% and that of the employees’ questionnaires was 65.4%. The effective recovery rate of the employees’ questionnaires is lower than that of the managers’ questionnaires, indicating that managers’ cooperation degree is higher than employees’ cooperation degree. The final analyzed sample comprised 314 groups. Table 1 shows the demographic data of the survey statistics. The characteristics of the enterprises are as follows: construction enterprises account for 61.1%, tobacco enterprises account for 8.9%, and other industrial enterprises account for 29.2%. Enterprises employing under 100 people accounted for 52.9%, enterprises with 100–300 people accounted for 17.2%, and enterprises with more than 300 people accounted for 29.2%. Of the managers, 80.3% were male, and 19.7% were female. In total 26.8% of the managers had a junior middle school education, and the remaining 73.2% had a high school education or above. Managers 20–30 years old accounted for 10.2% of the sample, those 31–40 years old accounted for 20.4%, those 41–50 years old accounted for 38.9%, and those 51–60 years old accounted for 30.6%. Of the employees, 75.5% were male, and 24.5% were female. In total, 38.2% of the employees had a junior middle school education, and the remaining 51.6% had a high school education or above. Employees aged 20–30 accounted for 26.8% of the sample, those age 31–40 accounted for 27.1%, those aged 41–50 accounted for 32.2%, and those aged 51–60 accounted for 14%. The gender ratio of managers and employees participating in the questionnaire survey includes more males than females because of the particularities of the construction, tobacco, and other industries.

**TABLE 1:** Descriptive statistics of demographic variables.

Classification		Number of people	Proportion: %
Form of business	Tobacco	14	8.9
enterprise	Construction	96	61.1
	Other	47	30
The scale of the	Less than 100 people	83	52.9
enterprise	From 100 to 300 people	27	17.2
	More than 300 people	47	29.9
Gender of	Male	126	80.3
management	Female	31	19.7
Age of	20–30	16	10.2
management	30–40	32	20.4
	40–50	61	38.9
	50–60	48	30.6
Educational qualifications of the	Junior high school and below	0	0
management	High school and technical secondary school	42	26.8
	College degree or above	115	73.2
The gender of the	Male	237	75.5
employee	Female	77	24.5
The age of the	20–30	84	26.8
employee	30–40	85	27.1
	40–50	101	32.2
	50–60	44	14.0
Employee’s degree	Junior high school and below	32	10.2
	High school and technical secondary school	120	38.2
	College degree or above	162	51.6

### Measurement of Variables

To ensure the rationality of this study and the excellent reliability and validity of the scales in the questionnaire, all variables in this paper are from the standard scales that have been empirically tested and matured by previous scholars. To ensure the rationality of this study and the good reliability and validity of the scales in the questionnaire, all variables in this paper are from the standard scales that have been empirically tested and mature by previous scholars. The selection of management psychological capital scale mainly refers to the western scholar Luthans et al. (2006) and Chinese scholar Ke et al. (2009a) to develop scales with different cultural characteristics; The selection of the scale of organizational emotional ability mainly refers to the scale developed by Akgün et al. (2009). The employee safety performance scale mainly refers to the scale developed by Indian scholar Vinodkumar and Bhasi (2010) and Chinese scholar Gao et al. (2015). The main variables in the questionnaire are psychological management capital, organizational emotional capability, and employee safety performance. In addition to the above variables, the questionnaire also includes the background information of the investigated enterprises, managers, and employees. Culture in different countries all have their own unique value systems, and cultural differences can cause individuals to generate heterogeneous feelings or perceive the same emotions (Chin et al., 2022). Moderation is Chinese people’s core cultural and psychological traits (Li et al., 2021). In addition to the control variables, the questionnaire is measured by a six-point Likert scale to avoid the impartiality of moderation and compromise, where one represents very inconsistent and six represents very consistent. The specific measurement scale design is shown in Table 2.

**TABLE 2:** Variable measurement items.

Variable	Label	Measure the question item
Psychological capital	PC1	I can do my current job.
	PC2	I love challenging tasks and desire success.
	PC3	I can objectively understand their shortcomings, humbly learn from the strengths of others.
	PC4	I can respect my leaders, colleagues, and subordinates and understand the shortcomings and mistakes of others.
	PC5	I keep my promises, listen to important decisions, and encourage others to make more comments.
	PC6	When meeting with management, I was confident in stating things within the scope of my work.
	PC7	I believe I can analyze long-term problems and find a solution.
	PC8	I can always keep a clear mind in adverse or favorable situations and important moments or occasions.
	PC9	At the moment, I am achieving the work goals that I have set for myself.
	PC10	At work, I will solve the problems anyway.
	PC11	I feel like I can handle many things simultaneously in my current work.
	PC12	At work, I usually expect the best when something is uncertain.
	PC13	The problems at work, I think, are short and can be solved and do not easily lead to frustration or despair.
	PC14	I am optimistic about what will happen in the future of my work.
Organize emotional abilities	OEA1	Our ability brings hope to our employees.
	OEA2	Our enterprise managers encourage a positive and enthusiastic treatment of the work.
	OEA3	Our company encourages our employees to express their opinions and emotions freely.
	OEA4	Our company manages its employees rigorously.
	OEA5	Our business can tolerate problems with our employees because of innovation.
	OEA6	Our employees can understand what others feel.
	OEA7	Our employees communicate closely with each other.
	OEA8	Employees in our company can understand their feelings without directly sharing their experiences.
	OEA9	The employees in our business are together because they have common interests, the most important of which are emotional bonds.
Safety performance	SP1	I will use the necessary safety equipment in strict accordance with the relevant regulations.
	SP2	I follow the correct security rules and procedures at work.
	SP3	I only work if I am sure it is safe.
	SP4	Sometimes I don’t follow the correct workflow because of a lack of time or familiarity with the work.
	SP5	I consciously participate in safety training, take the initiative to understand security knowledge or information.
	SP6	If I find any security-related issues in my company, I always point them out to management.
	SP7	I encourage my colleagues to work safely and proactively help them do their safety work well.
	SP8	I volunteer for tasks or activities that will help improve safety in the workplace.

#### Psychological Capital

Compared with Western culture, which emphasizes individual freedom and independence, Chinese culture pays more attention to managing interpersonal relationships, significantly impacting life and work. The research object of this paper is mainly Chinese enterprises. Therefore, considering the cross-cultural background, this paper considers the differences between Chinese and Western cultures in measuring psychological management capital and combines two Chinese and Western culture scales in selecting a psychological capital scale: (1) Western scholar Luthans et al. (2006) developed a scale with four dimensions: self-confidence, resilience, optimism, and hope. (2) Chinese scholar Ke et al. (2009a) developed an 8-dimensional scale of self-confidence, courage, optimism, ambition, tenacity, modesty, honesty, stability, tolerance, forgiveness, respect and comity, gratitude, and dedication. Examples of the 14 items include “I like challenging tasks and am eager for success” and “I can objectively understand my shortcomings, humbly learn from the strengths of others.”

#### Organizational Emotional Capability

Organizational emotional capability was measured by referring to the scale developed by Akgün et al. (2009) with nine items, such as “our company’s managers encourage positive and enthusiastic work” and “our company encourages employees to express their opinions and emotions freely.”

#### Safety Performance of Employees

In this study, the safety performance of employees was mainly measured with the scale developed by Vinodkumar and Bhasi (2010), translated by Gao et al. (2015) and is measured with eight questions: “I will strictly use necessary safety equipment under relevant regulations” and “I follow correct safety rules and procedures at work.”

#### Control Variables

This paper considers the potential impact of variables such as gender, age, and educational background of managers and employees on the research model. It sets the above variables as control variables. Management gender was denoted by MG, 1 = female, and 2 = male; Age was recorded as MA, 1 = under 20–30 years old, 2 = 31–40 years old, 3 = 40–50 years old, and 4 = 51–60 years old. Degree was recorded as MD, 1 = junior high school or below, 2 = senior high school or technical secondary school, and 3 = junior college or above. The gender of employees was recorded as EG, 1 = female, and2 = male. Age was recorded as EA, 1 = under 20–30 years old, 2 = 31–40 years old, 3 = 40–50 years old, and 4 = 51–60 years old. Education was recorded as EE: 1 = junior high school or below, 2 = senior high school or technical secondary school, and 3 = junior college or above.

#### Analysis Methods

The questionnaire in this paper was completed by the respondents through self-evaluation. Managers used the psychological capital scale for self-evaluation, and employees used the organizational emotional capability and safety performance scale. This study uses SPSS 26.0, SPSSAU, and AMOS24.0 to analyze the data. (1) Reliability analysis and exploratory factor analysis were carried out to verify the reliability and accuracy of the scale. (2) AMOS24 was used for further confirmatory factor analysis to scale for convergent validity and differentiate validity. (3) SPSS 26.0 and SPSSAU were used to conduct online analysis and hierarchical regression analysis on the sample data. (4) The process program embedded in SPSS 26.0 was used to test the mediation effect.

### Reliability and Validity Test

#### The Reliability

Before hypothesis testing, Cronbach’s α coefficient was used to test the reliability of the scale used. As shown in Table 3, the Cronbach’s α coefficients of managers’ psychological capital, organizational emotional capability, and employee safety performance are 0.965, 0.891, and 0.937, respectively. The reliability coefficients of psychological management capital, organizational emotional capability, and employee safety performance are more significant than 0.8, indicating that each scale has high internal consistency and good reliability (Eisinga et al., 2013).

**TABLE 3:** The reliability of variables.

Variable	Cronbach’ α coefficients	Number of terms
Psychological capital	0.965	14
Organize emotional abilities	0.891	9
Safety performance	0.937	8

#### Validity

In this paper, SPSS 26.0 is used to conduct exploratory factor analysis on the sample data using the Kaiser–Meyer–Olkin (KMO) test analysis method and Bartlett test of sphericity. The results show that the KMO value is 0.966, and the Bartlett test of sphericity is significant at the 0.000 level, indicating that the sample data are suitable for factor analysis.

In this paper, AMOS24 was also used to conduct confirmatory factor analysis (CFA) on the three factors and 31 analysis items of psychological management capital, organizational emotional capability, and employee safety performance. The necessary sample size was determined to be 314, which is more than ten times the number of analyzed items. The standard load coefficients of 14 psychological capital items are 0.797, 0.872, 0.758, 0.600, 0.817, 0.794, 0.758, 0.918, 0.834, 0.893, 0.842, 0.839, 0.906, 0.882, respectively. The standard load coefficients of the eight safety performance items are 0.920, 0.915, 0.912, 0.324, 0.872, 0.857, 0.912, and 0.892, respectively. The standard load coefficients of the 9 items of organizational emotional ability were 0.848, 0.881, 0.866, 0.496, 0.594, 0.763, 0.778, 0.484, and 0.714, respectively. The results showed that the standardized load coefficient of 30 items in the questionnaire were all greater than 0.4, and one item was 0.324. However, considering that the Cronbach α coefficient of the whole population does not change much after this item is deleted, the existence of this item in validity test is beneficial to explain the influence of factor 2. Only the loading coefficients of the two factors are small, which has little influence on the overall data analysis. In addition, this paper uses a relatively mature scale verified by empirical research, so it chooses to retain this item. As shown in Table 4, the average variance extractions (AVEs) of management psychological capital, organizational emotional capability, and employee safety performance were 0.666, 0.510, and 0.674, respectively, all greater than 0.5. The composite reliability (CR) values were 0.965, 0.941, and 0.895, respectively, all higher than 0.7, indicating that the data used in this analysis have good aggregation validity (Chung et al., 2004).

**TABLE 4:** Test of convergent validity test.

Variable	AVE	CR
Psychological capital	0.666	0.965
Organize emotional abilities	0.510	0.895
Safety performance	0.674	0.941

In this paper, AMOS24 was used to conduct confirmatory factor analysis on managers’ psychological capital, organizational emotional capability, and employees’ safety performance to test the discriminative validity. As shown in Table 5, for managers’ psychological capital and organizational emotional capability, the square root values of AVE were higher than the maximum absolute correlation coefficient between factors, indicating that they have good discriminative validity. For employee safety performance, its square root value of AVE is 0.821, which is indicating that it has good discriminant validity. The AVE’s square root of the three factors is greater than the correlation coefficient between factors, indicating that all variables in this study have good discriminant validity.

**TABLE 5:** Test of discrimination validity.

Variable	1	2	3
1. Psychological capital	0.816		
2. Organize emotional abilities	0.612	0.714	
3. Safety performance	0.744	0.634	0.821

## Analysis of Results

### Descriptive Statistical Analysis

Table 6 presents the means, standard deviations, and correlation coefficients of the variables, and from the data in the table, it can be concluded that psychological management capital and employee safety performance are significantly and positively correlated (*r* = 0.744, *p* < 0.01); management psychological capital and organizational emotional capability are significantly and positively correlated (*r* = 0.612, *p* < 0.01); organizational emotional capability and employee safety performance are significantly positively correlated (*r* = 0.634, *p* < 0.01); hypotheses H1∼H3 were initially tested.

**TABLE 6:** Means, standard deviations, and correlation coefficients of variables.

Variables	*M*	*SD*	1	2	3
1. Psychological capital	4.850	1.119	1		
2. Organize emotional ability	4.430	1.137	0.612**	1	
3. Safety performance	4.694	1.331	0.744**	0.634**	1

Notes: n = 314. The constant terms are omitted; *significant at p < 0.1; **significant at p < 0.05; and ***significant at p < 0.001.

### Hypothesis Test

This study applied SPSS 26.0 software to perform hierarchical regression on independent and dependent variables and verified hypotheses H1, H2, and H3. The multicollinearity test found that the VIF values in the model are all less than 5, which means that there is no collinearity, and the DW value is near the number 2, indicating that the model does not have autocorrelation. The model fits well. In each model in Table 7, step 1 represents the influence of the control variable on the dependent variable, and step 2 represents the influence of the independent variable on the dependent variable.

**TABLE 7:** Regression analysis of the relationship between management psychological capital, Organizing emotional capacity, and employee safety performance.

	Dependent variable						
	Model 1		Model 2		Model 3		Model 4
	Safety performance	Safety performance	Organizing emotional capacity	Organizing emotional capacity	Safety performance	Safety performance	Safety performance
**Control variables**	**Step 1**	**Step 2**	**Step 1**	**Step 2**	**Step 1**	**Step 2**	**Step**
MG	–0.009	–0.066	–0.077	–0.117	–0.009	0.049	0.036
MA	–0.06	0.008	–0.13	–0.083	–0.06	0.038	–0.093
MD	–0.037	–0.099	0.027	–0.016	–0.037	–0.057	–0.059
EG	–0.128	–0.08	–0.023	0.01	–0.128	–0.111	–0.084
EA	0.044	–0.028	0.137*	0.087	0.044	–0.06	0.076
EE	0.155	0.048	–0.007	–0.082	0.155	0.161	–0.026
**Independent variable**							
Psychological capital		0.887**		0.616**			0.673**
Organizing emotional capacity						0.754**	0.347**
*R* ^2^	0.01	0.557	0.028	0.39	0.01	0.413	0.611
Adjusted *R*^2^	–0.009	0.547	0.009	0.376	–0.009	0.4	0.601
*F* value	0.536	55.017	1.496	27.958	0.536	30.774	59.844

Notes: n = 314 The constant terms are omitted; *significant at p < 0.1; **significant at p < 0.05; and ***significant at p < 0.001.

First, the influence between management’s psychological capital and employee safety performance was analyzed. As shown by step 1 in model 1 of Table 7, taking the gender, age and other control variables of managers and employees as independent variables, and employee safety performance as the dependent variable, regression analysis found that the coefficients of the control variables were not significant, indicating that the control variables had little effect on the regression results of employee safety performance. The regression results of step 2, adding psychological capital as an independent variable based on step 1, after controlling for variables such as gender and age of managers and employees, showed that managers’ psychological capital had a significant positive effect on employee safety performance (β = 0.887, *p* < 0.01), which verified H1.

Second, the influence effect between the psychological capital of managers and the emotional ability of their organizations was analyzed. As shown by step 1 of model 2 in Table 7, taking control variables such as gender and age of managers and employees as independent variables, and organizational emotional capability as the dependent variable, the regression analysis found that employees’ age had a positive influence on organizational emotional capability (β = 0.137, *p* < 0.05), and the rest of the control variables’ coefficients were not significant, indicating that all the control variables except employee age had little effect on the regression results of organizational emotional capability; adding the independent variable psychological capital based on step 1 to get step 2, after controlling for gender and age of managers and employees, the results show that managers’ psychological capital has a significant positive impact on organizational emotional capability (β = 0.616, *P* < 0.01), which verifies H2.

Third, the relationship between organizational emotional capability and employee safety performance was analyzed. As shown by step 1 of model 3 in Table 7, the regression analysis was conducted with gender, age, and education of managers and employees as independent variables and employee safety performance as the dependent variable, and it was found that the coefficients of the control variables were not significant, indicating that the control variables had little effect on the regression results of employee safety performance. Step 2 is obtained by adding independent variables of psychological capital based on step 1. The results show that, after controlling variables such as gender and age of managers and employees, the organizational emotional capability has a significant positive impact on employee safety performance (ß = 0.754, *p* < 0.01), which verifies H3.

Fourth, the mediating effect of organizational emotional capability was analyzed by constructing model 4 with managers’ and employees’ gender, age, and education as control variables, managers’ psychological capital and organizational emotional capability as independent variables, and employees’ safety performance as the dependent variable. Based on the criterion of the mediating effect, it was found that organizational emotional capability played a partially mediating role in the relationships among managers, psychological capital, and employee safety performance, thus validating hypothesis H4.

The process plugin set the confidence interval to 95% and 5000 bootstrapping samples to further test the mediating effect of managers, psychological capital, and employee safety performance. To estimate the mediating effect of organizational emotional capability more accurately, this study applied the bootstrap method in SPSS. The 95% confidence interval calculated by bootstrap sampling is not significant if it contains 0, indicating no significant effect between the variables; conversely, it is significant. As shown in Table 8, the confidence interval of the total effect of managers’ psychological capital on employees’ safety performance is 0.797–0.977. When the gender and age of managers and employees were controlled, the confidence interval of the direct effect of managers’ psychological capital on employees’ safety performance was 0.57–0.78. The confidence interval of the mediating effect of organizational emotional capability is 0.13–0.31. The above three variables do not contain 0, such indicating that the total effect, direct effect and intermediary effect among variables are significant. It also shows that managers’ psychological capital can not only directly and significantly affect employees’ safety performance. Moreover, organizational emotional capability can positively influence employee safety performance through the mediating effect. Without the intermediary variables, the regression coefficient of the manager’s psychological capital on employee safety performance is 0.887; that is, the total utility value is 0.887. When the intermediary variables are included, the regression coefficient is 0.673; that is, the direct utility value is 0.673. The intermediary utility value of dynamic organization in the manager’s psychological capital on employee safety performance is 0.214; that is, the indirect effect value is 0.214. The direct effect and the intermediary effect account for 75.89 and 24.11% of the total effect, respectively. Manager’s psychological capital plays a mediating role in the influence of organizational emotional capability on employee safety performance; that is, it verifies Hypothesis H4 again.

**TABLE 8:** Decomposition of the total effect, direct effect, and mediating effect.

	Effect	BootSE	BootLLCI	BootULCI	Effectiveness ratio
Total effect	0.887	0.046	0.797	0.977	
Direct effect	0.673	0.054	0.567	0.779	75.89%
Intermediary effect	0.214	0.047	0.125	0.312	24.11%

## Research Conclusion

### The Influence Mechanism of Managers’ Psychological Capital on Employee Safety Performance

This paper investigates the data of construction and tobacco companies in China and selects a psychological capital scale with local characteristics. It explores the influence mechanism of managerial and psychological capital on employee safety performance from organizational emotional capability. The research results verify the following four hypotheses. H1: The psychological capital of managers significantly impacts employee safety performance; good psychological capital not only makes it easy for employees to accept the management of their leaders and hold positive attitudes toward the measures taken by their managers but also motivates employees to be willing to add more positive behaviors that conform to the rules. Managers with higher levels of psychological capital have strong willpower, do not become anxious in the face of challenges at work, believe that there are multiple solutions, have optimistic expectations about the outcome of challenges, and have the tenacity to persistently solve problems, which often have a positive impact on employee safety performance. H2: Managerial psychological capital has a significant impact on the emotional capacity of organizations; psychological management capital has the qualities of optimism, hope, self-efficacy, and resilience. Managers with high levels of psychological capital can communicate hope, joy, and positive emotions within their organizations. Managers with high self-efficacy are calm at work, dare to accept challenges, and can instill an emotional ability of “trial and error risk-taking” into the organization. H3: Organizational emotional capability has a significant impact on employee safety performance. The organization encourages employees to actively express their emotions, creates an atmosphere of mutual assistance, allows employees to communicate actively, encourages members to establish the same vision as the organization, brings hope and happiness to employees at work, and helps them achieve achievements. In addition, according to the theory of emotional events, emotional responses can significantly promote performance. Due to the mutual influence of organizational and individual emotions, organizational emotions are a valuable resource in the development process of an enterprise and motivate organizational development. Therefore, safety performance is closely related to the organizational performance of enterprises. H4: Managers’ psychological capital positively affects employees’ safety performance through the mediating effect of organizational emotional capability. Managers’ psychological capital is a psychological state that managers show at work, and it is a critical factor that affects employees’ behavior. The psychological capital of managers influences team members through positive emotional expression, allowing them to experience more positive emotions, affecting the emotional state of employees in the organization, and then having a positive impact on employees’ safety compliance behavior and participation behavior. Based on empirical analysis and focused on employee safety performance, this paper verifies that managers’ psychological capital directly affects performance through hierarchical regression and confidence interval estimation. In addition, managers’ psychological capital indirectly affects employee safety performance through the intermediary variable of organizational emotional capability.

### Practical Insights

Starting from the influence mechanism of managers’ psychological capital on employees’ safety performance, to improve enterprises’ safety performance in the production process and reduce the occurrence of accidents, this paper puts forward the following management practice suggestions:

First, enterprises should pay attention to cultivating managers’ psychological capital. Psychological capital is the promoter and catalyst of individuals’ positive working attitudes and behaviors and is essential for effective operation and long-term development (Chen et al., 2017). During the development process of construction and other industrial enterprises, management workers should reshape their psychological capital. By improving the psychological capital of managers, managers can set a good example for employees in practical work, increase employees’ safety compliance behaviors, and enhance enthusiasm for safety participation, thereby affecting the improvement in employees’ safety performance. The specific methods to improve the psychological capital ability of managers are as follows: (1) Psychological capital assessment items should be included in the selection process of managers. The score should be used as an essential reference basis for selecting managers; especially when selecting managers in safety management-related positions, the weight of the psychological capital score should be increased. (2) In the training process of managers, courses related to psychological capital cultivation should be added so that managers can understand the meaning of psychological capital and improvement methods and consciously improve their psychological capital ability in daily management practice. (3) Monitoring and supervising the mental health of managers is over time. Regular assessment and counseling should be conducted to avoid safety problems caused by managers’ psychological capital ability decline.

Second, companies should attach importance to the cultivation of organizational emotional capability. Studies on organizational emotion show that although emotion is an internal psychological experience, it has the dual characteristics of psychology and sociology. It can act on the organization just as it does on employees, thus affecting employees’ organizational assumptions or basic judgments about the organization (Hareli and Rafaeli, 2008). Currently, organizational emotional capability is a concrete manifestation of the internal emotional mental state of organizations and an essential resource within organizations. It is affected by multiple prefactors, such as leadership type and team effectiveness, while also having a meaningful impact on organizational performance and other outcome variables (Li et al., 2019). In this study, organizational emotional capability plays an intermediary role in managers’ psychological capital on employee safety performance. Based on this, in practice, while companies attach importance to employee safety management, they also need to pay attention to the development of organizational emotional capability. The specific measures are as follows: (1) Create a positive and good organizational safety environment in the working environment of managers and employees and maintain the order within the organization based on a relaxed and happy mood. (2) Encourage employees to have the spirit of innovation and practice in their work, dare to use new methods, try mistakes, and be willing to take on challenges. (3) Encourage employees to express their true feelings, create a harmonious organizational environment and form free communication channels. (4) Encourage mutual understanding and respect among employees to feel the significance and value of safe work. (5) Create a positive organizational culture. During the creation process, the organization’s employees’ emotions will be deepened to influence and regulate employees’ safety compliance behaviors at work and consciously encourage them to participate in safer training and publicity activities. It effectively improves the safety performance of the entire organization.

### Theoretical Significance

This research makes theoretical contribution to the study of organizational emotional capability by studying the influence mechanism of managers’ psychological capital on employee safety performance. First, regarding the psychological capital of managers and employee safety performance, previous scholars’ research has mainly focused on the impact of employees’ psychological capital on employee behavior, attitudes, and organizational performance. This study focuses on the safety performance of employees in high-risk industrial enterprises and explains the influence mechanism by which psychological capital affects safety performance. Research has found that managers with higher levels of psychological capital influence the safety performance of employees through the effect of role models. Managers with higher psychological capital inject hope into their organizations, bring joy to employees, create a positive working environment, and encourage employees to reasonably express their true feelings. Second, organizational emotional capability act as an intermediary variable. Enterprises should experience and feel the emotions of organization members and enhance their sense of belonging. These measures are conducive to effective communication between managers and employees and between employees and enhance employees’ safety compliance behaviors and participation behaviors, thereby affecting their safety performance. In addition, choosing organizational emotional capability as an intermediary variable in research can further enrich the theory of the impact of managers’ psychological capital on employee safety performance.

### Research Limitations and Suggestions for Future Research

This paper conducts an empirical analysis of the impact mechanism on the psychological capital of managers and employee safety performance in construction and other industrial enterprises. Due to the constraints of conditions, the current study has the following shortcomings. (1) This study analyzes only the overall three dimensions of managers’ psychological capital, organizational emotional capability, and employee safety performance and does not subdivide the above three variable dimensions, such as carrying out a subdimension exploration of self-efficacy, optimism, hope, and resilience in demission of psychological capital. (2) The data used in the study are mainly cross-sectional data, which cannot inform the causal relationship between variables. Future studies will consider using a longitudinal survey method of interval time to verify the causal relationship between variables further.

In future research, first, the psychological capital of managers should be divided into subdimensions, and its influence mechanism can be further explored from the internal effect of psychological capital. Second, the research on the impact of safety performance can also be divided into two dimensions of safety compliance behavior and safety participation behavior for empirical research, and the influence mechanism of antecedent variables on safety performance can be analyzed in various dimensions. Finally, moderating variables are introduced to explore the various mechanisms by which managers’ psychological capital influences employees’ safety performance in various scenarios.

## Data Availability Statement

The raw data supporting the conclusions of this article will be made available by the authors, without undue reservation.

## Ethics Statement

Ethical review and approval was not required for the study on human participants in accordance with the local legislation and institutional requirements. Written informed consent from the patients/participants or patients/participants legal guardian/next of kin was not required to participate in this study in accordance with the national legislation and the institutional requirements.

## Author Contributions

XZ, YT, XJ, and HL organized the collection and preparation of data. CP and KX performed the statistical analyses, wrote the first draft of the manuscript, and made changes to the manuscript during the interactive review stage. All authors contributed to the conception and design of the study, read, and edited the manuscript, and suggested improvements at several stages during the preparation and revision of the manuscript.

## Conflict of Interest

HL is employed by OCT Yunnan Cultural Investment Group Co., Ltd. The remaining authors declare that the research was conducted in the absence of any commercial or financial relationships that could be construed as a potential conflict of interest. The reviewer JS declared a shared affiliation, with no collaboration, with one of the authors, KX to the handling editor at the time of the review.

## Publisher’s Note

All claims expressed in this article are solely those of the authors and do not necessarily represent those of their affiliated organizations, or those of the publisher, the editors and the reviewers. Any product that may be evaluated in this article, or claim that may be made by its manufacturer, is not guaranteed or endorsed by the publisher.
